# Impact of Resection Volume/Stapler Firings-Ratio on Perioperative Complications and Weight Loss After Laparoscopic Sleeve Gastrectomy

**DOI:** 10.1007/s11695-020-04870-0

**Published:** 2020-07-29

**Authors:** Andrea Della Penna, Jessica Lange, Rami Archid, Felix Hönes, Alfred Königsrainer, Markus Quante

**Affiliations:** Department of General, Visceral and Transplant Surgery, University Hospital Tübingen, Hoppe-Seyler-Strasse 3, 72076 Tübingen, Germany

**Keywords:** Sleeve gastrectomy, Stapler firings, Staple line leaks, Gastric resection volume, Weight loss

## Abstract

**Background:**

Major postoperative morbidity after laparoscopic sleeve gastrectomy (LSG) is often related to staple line leaks (SLL). Of note, a recent study suggested a central role of the absolute numbers of stapler firings as a predictive factor for postoperative morbidity due to SLL. In addition, a larger gastric remnant volume could be responsible for lower weight loss after LSG, and nevertheless, the gastric resection volume (GRV) is strictly related to the residual volume.

**Methods:**

Prospectively, collected data of 384 consecutive patients with complete follow-up at 12 months after LSG at our institution were retrospectively analyzed. Patients were stratified according to three different variables (i.e., number of stapler firings, GRV, and GRV/stapler firings-ratio), and respective impact on postoperative complications and weight loss was analyzed.

**Results:**

High absolute number of stapler firings was linked to increased intraoperative and postoperative bleeding and prolonged hospitalization, but was not associated with SLL, transfusion rate or revisional procedures. Absolute GRV showed no impact on both complications and outcome after LSG. Interestingly, higher ratio of GRV/stapler firings was not only linked to decreased intraoperative bleeding and shorter hospital stay but also to higher Excess Body Mass Index Loss (EBMIL) at 12 months after LSG.

**Conclusions:**

Here, we introduce GRV/stapler firings-ratio as a simple predictive factor for identifying patients at risk for postoperative complications and impaired weight loss that is superior compared with absolute number of stapler firings or GRV alone.

## Introduction

Morbid obesity has become a global epidemic. This is reflected by recent WHO data reporting > 650 million people worldwide being obese in 2016, finally accounting for 13% of the entire adult global population [1]. In parallel, the global number of bariatric surgery procedures performed per year is steadily increasing as well. In 2016, the International Federation for the Surgery of Obesity and Metabolic Disorders (IFSO) reported > 685.000 bariatric procedures being performed worldwide. Among these bariatric procedures, laparoscopic sleeve gastrectomy (LSG) represents the top utilized bariatric procedure currently accounting for > 50% of primary surgical bariatric procedures registered worldwide in 2016 [2, 3].

LSG was first described as one step of the biliopancreatic diversion with duodenal switch and subsequently as the first stage of multi-step procedures for the treatment of super-obese patients, in order to reduce perioperative risk and complications [4–6]. After initial experiences, a wide number of studies reporting on LSG as a stand-alone procedure resulting in substantial weight loss have been published. Consequently, LSG was approved as a primary bariatric procedure in a position statement of the American Society for Metabolic and Bariatric Surgery (ASMBS) in 2010 [7]. During the past 10 years, the number of LSG procedures performed worldwide has been steadily increasing. With growing data and experience, the LSG procedure could be linked to several advantages such as shorter operation time, lack of enteric anastomosis and malabsorption, no or low risk of ulceration and internal hernia, lower rates of dumping syndrome, better patient’s acceptance, feasibility to be converted into multiple other bariatric procedures, maintenance of gastrointestinal continuity with feasibility of endoscopic assessment, lower grade of technical difficulty, and also a lower rate of perioperative complications when compared with the bariatric “gold-standard” of laparoscopic Roux-en-Y gastric bypass (LRYGB) [8, 9].

Commonly reported complications following LSG are bleeding, strictures, and staple line leaks, with the latter one still representing a major matter of concerns [10]. Considering only primary bariatric procedures, the leakage rates reported after LSG are varying from 1 to 3% [10, 11]. However, leakage rate can significantly increase up to 10% in cases when LSG is performed as a revisional procedure [12, 13]. Since major postoperative morbidity after LSG is often related to SLL, many efforts have been made to better understand pathophysiological and technical aspects related to its onset. In more detail, SLL can be divided into two major categories of pathogenetic causes, i.e., ischemic or mechanical [14]. For the latter one, technical aspects related to stapler misfiring and the type of cartridge used were widely discussed in a recent review by Iossa et al., finally leading to the statement that appropriate cartridge color should be used based on different stomach wall thickness [14]. Another recent work by Major et al. suggested a central role of the absolute numbers of stapler firings as a predictive factor for postoperative morbidity and SLL [15]. The authors demonstrated that a high absolute number of stapler firings during the procedure should alert the surgeon for an increased risk of postoperative morbidity, but the hypothesized mechanism remains unclear [15].

Among the technical aspects predicting the success of the LSG in terms of weight loss, the use and size of a bougie to calibrate the sleeve has been widely appreciated. Many studies have been carried out in order to find out the most effective bougie size linked to reduction of leakage rates but without affecting the results in terms of weight loss [14]. Indeed, a larger gastric remnant volume could be responsible for lower weight loss after LSG, and nevertheless, the gastric resected volume (GRV) is strictly related to the residual volume [16]. Even though some authors strictly exclude possible impact of GRV on weight loss after LSG [17, 18], a recent prospective study by Sista et al. demonstrated that the GRV is affecting outcomes after LSG, therefore being a predictive factor for weight loss and reduction of comorbidity [19]. Recently, a prospective study by Kim et al. could associate preoperative stomach volume assessed by three-dimensional computed tomography with visceral fat volume and BMI, thus pointing out towards a more tailored therapeutic approach in bariatric surgery based on preoperative variables [20].

Taken together, simple predictive factors for identifying patients at high risk for SLL or impaired weight loss would be highly desirable. With the background of the cited literature, we thus retrospectively reviewed patients undergoing LSG at our center. Here, we hypothesized that surgical technique as reflected by the number of stapler firings and the GRV will impact both postoperative complications and 12-month weight loss results after LSG.

## Materials and Methods

### Patients and Inclusion Criteria

A retrospective analysis of our prospectively collected database of all patients undergoing bariatric procedures at the University Hospital of Tübingen, Germany, between January 2007 and November 2017 was performed. Patients undergoing combined bariatric procedures and other stand-alone surgical or endoscopic procedures besides LSG were excluded.

From the cohort of patients undergoing primary LSG procedures, those with complete clinical follow-up at 12 months after the procedure were selected for final analysis. Indication to LSG was given in all patients according to the German Guidelines for Obesity Surgery, and informed consent to surgical procedure was obtained from all patients.

### LSG Technique

The surgical procedure at our institution followed a standard technique as described by our group before [21]. In brief, a gastric tube was positioned along the minor gastric curvature, and the gastric sleeve was performed along the bougie using a 60-mm Ethicon Echelon Stapler (Ethicon Endo-Surgery Inc., Cincinnati, OH) with a bioabsorbable staple line reinforcement (GORE® SEAMGUARD® Reinforcement, W. L. Gore & Associates, Elkton, MD) and with sequential firings of linear green and blue GIA reloads. Next, the staple line was tested with methylene blue dye. The resected stomach was removed through the right flank trocar site. The volume of the resected stomach was measured on the back table in the operation room.

### Patient Stratification

Patients were divided into three groups based on the quartile distributions (group 1 ≤ 25; 25 < group 2 < 75; group 3 ≥ 75) to simplify data interpretation since the outcome can be described in terms of a relative risk between groups stratified according to the following variables:

I) Absolute number of staple firings used during the procedure
II) Absolute GRV
III) Calculated quotient between GRV and number of staple firings

### Statistical Analyses

Categorical variables are presented as absolute numbers and percentages, and the *X*^*2*^ test was used for comparison. Continuous variables were tested for normality using the Shapiro-Wilk test. All continuous variables were not normally distributed and thus presented as median values with ranges between lowest and highest value. Outcome measures were assessed across these three groups. Comparison between groups for continuous variables was performed using the Kruskal-Wallis test. Multivariate logistic regression models were performed for each of our outcome measures. These models were adjusted for age, gender, and preoperative BMI. Results were considered statistically significant if *P* values were < 0.05.

### Ethics

All procedures performed in the study were in accordance with the ethical standards of the institution and/or national research committee and with the 1964 Helsinki Declaration and its later amendments or comparable ethical standards. Informed consent for surgical treatment was obtained from all patients before the surgical approval. The study was approved by the local Ethics Review Committee (960/2018BO2).

## Results

**Fig. 1:**
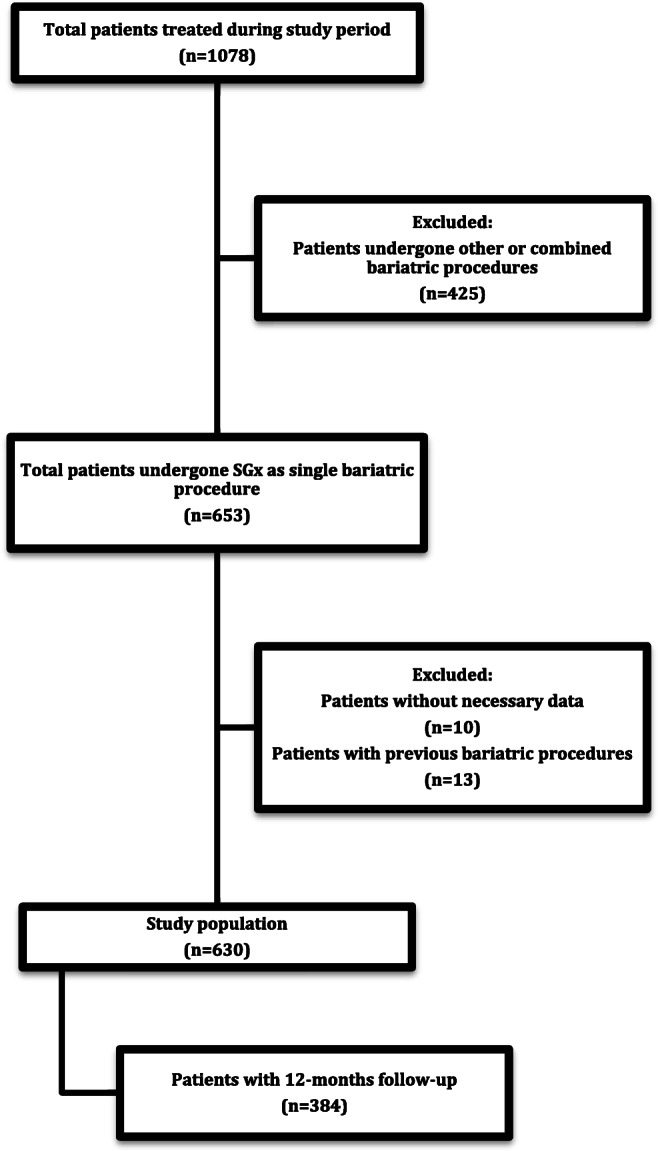
Study flowchart

During the study period, a total of 1078 patients underwent bariatric surgery at our institution. Among those, 653 patients were treated with LSG. After exclusion of patients that had previously undergone other bariatric procedures (*n* = 13) and those patients without necessary primary data for analysis (*n* = 10), a cohort of 630 patients undergoing LSG as a stand-alone bariatric procedure were identified. Of those patients, a total number of 384 patients had complete clinical follow-up at 12 months after the surgical procedure and were thus selected for final analysis (Fig. 1).

Median age of our patients at time of surgery was 43 years (range 18–68); 95 patients (24.7%) were male, and 289 patients (75.3%) were female. Median weight before surgery was 145 kg (range 82–238) with a median BMI of 51.1 kg/m^2^ (range 36.6–76.2). No major intraoperative complications (intraoperative transfusion, conversion to laparotomy, dead) occurred, and median blood loss during the procedure was 7.5 ml (range 0–1000). The median number of stapler firings during the procedure was 5 (range 4–11), the median GRV in the entire cohort was 875 ml (range 300–2100), and the resulting GRV/stapler firings-ratio showed a median value of 175 ml/stapler firing (range 27–400).

A total number of 7 (1.8%) staple line leaks occurred, 5 (1.3%) of them presenting as covered perforation and 2 (0.5%) as diffuse peritonitis in the early postoperative course. Postoperative bleeding occurred in 6 (1.6%) patients. For postoperative surveillance and management after LSG,120 (32%) of our patients were admitted to the intensive care unit (ICU); median hospital stay was 5 days (range 3–116). Of note, increasing experience during the 10-year observation period resulted in lower rates of ICU admission and earlier discharge from hospital over time.

### Stapler Firings Analysis

Perioperative complications and postoperative outcome of the three different quartile groups based on the absolute number of stapler firings during the LSG procedure are depicted in Table 1. A high absolute number of stapler firings were linked to significantly increased intraoperative blood loss, postoperative bleeding, and need for relaparoscopy (*P* < 0.001, *P* = 0.042, and *P* = 0.033, respectively). Furthermore, median ICU and hospital stay were also significantly prolonged in patients with high absolute numbers of stapler firings during LSG procedure (*P* < 0.001 each). However, there were no significant differences in regard to staple line leakage (*P* = 0.364) and need for blood transfusion (*P* = 0.675). Clinical results at 12 months after LSG did not reveal any significant differences in the distribution of median body mass index (BMI), EBMIL, and total weight loss (TWL).

**Table 1:** Staple firings analysis

	Stapler Group 1 (*N* = 78)	Stapler Group 2 (*N* = 276)	Stapler Group 3 (*N* = 30)	*P* value
Gender, M/F	6/72	72/205	18/12	< 0.001
Age, median (min-max) [years]	35.5 (18–67)	43 (18–67)	47 (21–68)	0.001
Weight preoperative, median (min-max) [kg]	135.5 (82–187)	146 (97–238)	164 (109–205)	< 0.001
BMI preoperative, median (min-max) [kg/m^2^]	50.69 (40.15–68.44)	51.5 (36.61–76.17)	50.66 (38.62–72.53)	0.382
Hospital stay, median (min-max) [days]	5 (3–8)	5 (3–53)	6 (5–116)	< 0.001
ICU stay, median (min-max) [days]	0 (0–2)	0 (0–39)	1 (0–41)	< 0.001
Bleeding intraoperative, median (min-max) [mL]	0 (0–400)	10 (0–1000)	20 (0–600)	< 0.001
Resected gastric volume, median (min-max) [mL]	800 (400–1500)	900 (350–2100)	1000 (300–1750)	0.003
Volume/stapler ratio, median (min-max) [mL/n° of cartridge]	200 (100–375)	166 (66–400)	142 (27–250)	< 0.001
Gastric leakage, n° (%)	0 (0)	6 (2.2)	1 (3.3)	0.364
Bleeding postoperative, n° (%)	0 (0)	4 (1.4)	2 (6.7)	0.042
Transfusion postoperative, n° (%)	0 (0)	2 (0.7)	0 (0)	0.675
Relaparoscopy, n° (%)	0 (0)	10 (3.6)	3 (10)	0.033
Weight at 1 year postoperative, median (min-max) [kg]	92.5 (43–147)	99.5 (55–170)	105 (68–165)	0.004
BMI at 1 year postoperative, median (min-max) [kg/m^2^]	34.41 (21.33–56.8)	34.82 (21.47–54.28)	34.85 (22.59–59.8)	0.797
EBMIL at 1 year postoperative, median (min-max) [%]	63.24 (21.91–123.45)	62.2 (18.04–122.12)	59.63 (26.79–114.09)	0.919
TWL at 1 year postoperative, median (min-max) [%]	30.29 (13.58–51.67)	32.2 (8.33–56.6)	30.02 (17.55–52.12)	0.641

Group 1, ≤ 4 staple firings; Group 2, 5–6 staple firings; Group 3, ≥ 7 staple firings

Three groups were stratified based on quartile distribution of absolute number of staple firings

### Gastric Resected Volume Analysis

Based on the quartile distribution of GRV values, three groups were identified and respective outcomes were compared. Here, only median ICU and median hospital stay were significantly prolonged in patients with low GRV (*P* = 0.048 and *P* = 0.004, respectively), while there was no impact of GRV on the occurrence of perioperative complications. When comparing weight loss results at 12 months after surgery, there was no impact of GRV on either BMI, EBMIL, or TWL (Table 2).

**Table 2:** Gastric resected volume (GRV) analysis

	Volume Group 1 (*N* = 104)	Volume Group 2 (*N* = 184)	Volume Group 3 (*N* = 96)	*P* value
Gender, M/F	10/94	40/144	45/51	< 0.001
Age, median (min-max) [years]	43.5 (19–68)	42.5 (18–67)	405 (18–65)	0.043
Weight preoperative, median (min-max) [kg]	139.5 (82–190)	145 (97–228)	156 (109–238)	< 0.001
BMI preoperative, median (min-max) [kg/m^2^]	51.27 (36.81–68.44)	51.34 (36.61–76.17)	51.09 (38.62–67.37)	0.999
Hospital stay, median (min-max) [days]	6 (3–116)	5 (3–53)	5 (3–11)	0.004
ICU stay, median (min-max) [days]	0 (0–41)	0 (0–39)	0 (0–3)	0.048
Stapler used, median (min-max) [n° of cartridge]	5 (4–11)	5 (4–9)	5 (4–10)	0.003
Bleeding intraoperative, median (min-max) [mL]	10 (0–1000)	5 (0–115)	5 (0–600)	0.052
Gastric leakage, n° (%)	1 (1)	5 (2.7)	1 (1)	0.454
Bleeding postoperative, n° (%)	2 (1.9)	2 (1.1)	2 (2.1)	0.768
Transfusion postoperative, n° (%)	1 (1)	1 (0.5)	0 (0)	0.639
Relaparoscopy, n° (%)	3 (2.9)	7 (3.8)	3 (3.1)	0.906
Weight at 1 year postoperative, median (min-max) [kg]	95 (43–146)	98 (55–165)	102 (68–170)	0.018
BMI at 1 year postoperative, median (min-max) [kg/m^2^]	34.96 (21.33–56.8)	35.29 (21.47–59.8)	34.26 (22.59–51.56)	0.683
EBMIL at 1 year postoperative, median (min-max) [%]	62.86 (18.04–123.45)	60.91 (26.79–122.12)	66.12 (31.05–114.09)	0.535
TWL at 1 year postoperative, median (min-max) [%]	30.57 (8.33–52.29)	31.84 (14.47–51.85)	32.87 (15.08–56.6)	0.440

Group 1, ≤ 700 ml; Group 2, 700–1100 ml; Group 3, ≥ 1100 ml

Three groups were stratified based on quartile distribution of GRV

### GRV/Stapler Firings-Ratio Analysis

After quotient calculation of GRV and absolute number of stapler firings during the LSG procedure, a final stratification into three groups based on the quartile distribution of GRV/stapler firings-ratio was performed. Here, intraoperative bleeding, ICU stay, and hospital stay were negatively linked to low GRV/stapler firings-ratio (*P* < 0.001 each). In addition, high GRV/stapler firings-ratio was significantly associated with favorable BMI and EBMIL at 12 months after LSG procedure (*P* = 0.034 and *P* = 0.047, respectively; Table 3).

**Table 3:** GRV/staple firings ratio subgroup analysis

	Ratio Group 1 (*N* = 89)	Ratio Group 2 (*N* = 203)	Ratio Group 3 (*N* = 92)	*P* value
Gender, M/F	16/73	43/160	36/56	0.001
Age, median (min-max) [years]	36 (22–68)	43 (18–67)	38 (18–65)	< 0.001
Weight preoperative, median (min-max) [kg]	145 (82–228)	143 (97–204)	149 (110–238)	0.037
BMI preoperative, median (min-max) [kg/m^2^]	52.94 (37.81–70.37)	50.61 (36.61–76.17)	51.04 (38.97–67.37)	0.074
Hospital stay, median (min-max) [days]	6 (3–116)	5 (3–53)	5 (3–11)	< 0.001
ICU stay, median (min-max) [days]	0 (0–41)	0 (0–39)	0 (0–1)	< 0.001
Bleeding intraoperative, median (min-max) [mL]	10 (0–800)	5 (0–1000)	3,5 (0–50)	< 0.001
Gastric leakage, n° (%)	1 (1.1)	5 (2.5)	1 (1.1)	0.611
Bleeding postoperative, n° (%)	0 (0)	6 (3)	0 (0)	0.066
Transfusion postoperative, n° (%)	0 (0)	2 (1)	0 (0)	0.408
Relaparoscopy, n° (%)	1 (1.1)	11 (5.4)	1 (1.1)	0.066
Weight at 1 year postoperative, median (min-max) [kg]	99 (43–165)	98 (55–157)	100 (63–170)	0.212
BMI at 1 year postoperative, median (min-max) [kg/m^2^]	35.88 (21.33–56.8)	34.85 (21.47–59.8)	34.17 (23.14–51.56)	0.034
EBMIL at 1 year postoperative, median (min-max) [%]	59.86 (18.04–123.45)	62.02 (26.79–122.12)	66.15 (29–111.26)	0.047
TWL at 1 year postoperative, median (min-max) [%]	30.5 (8.33–52.29)	31.75 (14.47–51.85)	33.12 (15.08–56.6)	0.328

Group 1, < 140 ml/staple firing; Group 2, ≥ 140 and ≤ 212 ml/staple firing; Group 3, > 212 ml/staple firing

Three groups were stratified based on quartile distribution of GRV/staple firings ratio

## Regression Model Analyses

We also performed regression model analyses for postoperative morbidity and weight loss outcome adjusted for baseline characteristics. Each odds ratio (OR) is adjusted for age, gender, and preoperative BMI and derived by separate logistic regression models. As a result, regression analyses confirmed significance of GRV/stapler ratio for postoperative morbidity in terms of intraoperative bleeding (OR = 2.35; confidence interval (CI), 1.41–3.93), ICU stay (OR = 2.72; CI, 1.63–4.55), and hospital stay (OR = 2.65; CI, 1.59–4.42). Along with our previous results, GRV/stapler ratio is increasing the overall predictive value of stapler firing alone. In contrast, GRV alone showed no significant impact on postoperative morbidity (Table 4). Regression analyses regarding postoperative weight loss could not confirm the previous significance level (data not shown). However, adjusted regression analysis revealed comparable odds ratios for GRV and GRV/stapler ratio in contrast to stapler firings alone. These results might point out towards increased impact of GRV alone on 12-month weight loss, while it had no significant impact on postoperative morbidity. Taken together, GRV/stapler ratio is increasing the overall predictive value of stapler firing alone, while GRV alone showed no significant impact.

**Table 4:** Regression model analyses

Adjusted odds ratios (OR) regarding hospital stay for Quartile 4 versus Quartiles 1–3 by subgroups		
Subgroup	Adjusted OR (95% CI)	*P* value
Stapler firings	5.02 (1.95; 12.95)	0.001
GRV in ml	0.90 (0.55; 1.46)	0.66
GRV/stapler ratio	2.65 (1.59; 4.42)	< 0.001
Adjusted odds ratios regarding ICU stay for Quartile 4 versus Quartiles 1–3 by subgroups		
Subgroup	Adjusted OR (95% CI)	*P* value
Stapler firings	11.92 (4.32; 32.86)	< 0.001
GRV in ml	1.09 (0.64; 1.84)	0.75
GRV/stapler ratio	2.72 (1.63; 4.55)	< 0.001
Adjusted odds ratios regarding intraoperative bleeding Quartile 4 versus Quartiles 1–3 by subgroups		
Subgroup	Adjusted OR (95% CI)	*P* value
Stapler firings	2.10 (0.91; 4.83)	0.08
GRV in ml	0.84 (0.51; 1.38)	0.49
GRV/stapler ratio	2.35 (1.41; 3.93)	0.001

Each OR is adjusted for age, gender, and preoperative BMI and derived by separate logistic regression models

## Discussion

As the major finding, we identified GRV/stapler firings-ratio as a simple predictive factor to identify patients at risk for postoperative complications and impaired weight loss that is superior compared with absolute number of stapler firings or GRV alone.

Over the last decade, laparoscopic sleeve gastrectomy has become the most commonly performed bariatric procedure, recently accounting for almost 50% of all primary bariatric procedures performed worldwide from 2014 to 2018 [3, 22]. Nevertheless, perioperative complications also occur after LSG and can be life-threatening for the affected patient [23, 24]. In particular, staple line leakage still represents the Achilles’ heel of the LSG procedure, and its incidence has been reported ranging from 1 to 3% [23]. Of note, SLL still represents the second most common cause of death after LSG among an overall reported postoperative mortality of 0.4% [25].

Therefore, many studies have focused on identification of perioperative factors predicting its onset. Of note, the association of multiple stapler firings and higher risk of anastomotic leakage has already been described in colorectal surgery [26]. Along the same lines, a recent study by Major et al. was the first to analyze the impact of the absolute number of stapler firings during LSG procedure on postoperative outcomes. In this cited study, the median number of stapler cartridges used during LSG procedure was 4 (range 3 to 8), and in multivariate logistic regression analysis, the absolute number of stapler firings was significantly related to a higher rate of postoperative complications [15]. In contrast, our results did not show any impact of the absolute number of stapler firings on the incidence of SLL. However, high absolute number of stapler firings was linked to increased intraoperative blood loss, postoperative bleeding, and prolonged hospital stay. Thus, our data provide further evidence that the higher number of stapler firings during LSG is an indicator of surgical technique reflecting intraoperative difficulties finally resulting in a higher rate of postoperative complications, although there was no association with the incidence of SLL.

Another central factor being discussed as a predictor of both postoperative complications and weight loss results after LSG is the GRV. Although its impact has been widely discussed, the results are mainly discordant, thus fueling an ongoing debate [16–19, 27]. Here, a recent prospective study suggested that GRV is impacting the outcomes subsequent to LSG during short-term and midterm follow-up while at the same time predicting the weight loss results in terms of percentage of excess weight loss (%EWL) and control of obesity-related metabolic complications [19]. Along the same lines, Weiner et al. reported that a GRV of < 500 ml seems to be a predictor of treatment failure or early weight regain [28], while another recent study could link GRV of > 1100 ml with significantly greater %EWL at 12 months compared with patients with a GRV of ≤ 1100 ml [27]. In contrast, we could not find an association between GRV and weight loss outcome in our patient cohort. As discussed before in a recent review, GRV tends to be variable according to preoperative patient characteristics and seems to be higher in patients having a higher preoperative BMI [29]. However, when we stratified our patients based on the quartile distribution of GRV values, all groups showed almost identical preoperative BMI (*P* = 0.999). In contrast, differences in preoperative weight were highly significant (*P* < 0.001). Thus, our results provide further evidence that the pure number of GRV is depending on preoperative patients´ characteristics. Taken together, simple extrapolation of weight loss based on GRV seems not reliable.

Since both variables, absolute number of stapler firings and GRV, are associated with preoperative weight in our patient cohort, we hypothesized that “internal normalization” by calculating a quotient of GRV and stapler firings—thus reflecting resected gastric volume per single stapler cartridge—might increase the overall predictive value. Therefore, we also stratified our patients into three groups based on the quartile distribution of GRV/stapler firings-ratio and compared respective outcomes. Strikingly, we were now able to demonstrate that the lower quartile of GRV/stapler firings-ratio (< 140 ml/cartridge) was linked to increased intraoperative bleeding and prolonged ICU stay and hospital stay, while the upper quartile of GRV/stapler firings-ratio (> 212 ml/cartridge) was associated with favorable loss of BMI and EBMIL at 12 months after surgery. As discussed before, current concepts were focusing either on stapler firings [15] or GRV [27] alone. However, both factors were variable in our patient cohort, thus limiting the predictive value. Here, calculating a quotient of GRV and stapler firings could reduce the variability by “internal normalization,” thus fostering the predictive value.

Therefore, we propose a novel and simple approach that might be helpful as a clinical tool for patient identification that warrants further prospective evaluation. We are aware that our study has inherent limitations due to the retrospective design and a relatively small number of patients. However, GRV/stapler firings-ratio might serve as a simple predictive factor to identify patients at risk for postoperative complications and impaired weight loss.

## Abbreviations

%EWL: Percentage of excess weight loss
BMI: Body mass index
CI: Confidence interval
EBMIL: Excess Body Mass Index Loss
GRV: Gastric resection volume
ICU: Intensive care unit
LRYGB: Laparoscopic Roux-en-Y gastric bypass
LSG: Laparoscopic sleeve gastrectomy
OR: Odds ratio
SSL: Staple line leaks
TWL: Total weight loss
